# Mapping the evidence and gaps of interventions for pediatric chronic pain to inform policy, research, and practice: A systematic review and quality assessment of systematic reviews

**DOI:** 10.1080/24740527.2020.1757384

**Published:** 2020-06-19

**Authors:** Kathryn A. Birnie, Carley Ouellette, Tamara Do Amaral, Jennifer N. Stinson

**Affiliations:** a Department of Anesthesiology, Perioperative and Pain Medicine, Foothills Medical Centre, University of Calgary, Calgary, Alberta, Canada; b Faculty of Nursing, McMaster University, Hamilton, Ontario, Canada; c Child Health Evaluative Sciences, The Hospital for Sick Children Peter Gilgan Centre for Research and Learning, Toronto, Ontario, Canada; d Lawrence S. Bloomberg Faculty of Nursing, University of Toronto, Toronto, Ontario, Canada

**Keywords:** pediatric, chronic pain, treatment, systematic review, policy, knowledge translation, evidence map

## Abstract

**Background**: Reviews in pediatric chronic pain often focus on only one intervention or population, making it difficult for policymakers and decision makers to quickly synthesize knowledge to inform larger-scale policy and funding priorities.

**Aims**: The aims of this study were to (1) create an evidence and gap map of interventions for pediatric chronic pain and (2) identify gaps between existing evidence and recently identified patient-oriented research priorities.

**Methods**: We performed a systematic review of English-language peer-reviewed systematic reviews or clinical practice guidelines of pediatric chronic pain intervention published in the past 20 years. Database searches of Medline, Embase, PsycINFO, Web of Science, CINAHL, and SCOPUS were conducted inclusive to June 3, 2019. Review quality was assessed using the AMSTAR-2.

**Results**: Of 4168 unique abstracts, 50 systematic reviews (including 2 clinical practice guidelines) crossing diverse pediatric chronic pain populations and intervention settings were included. One third were rated high quality, with half rated low to critically low quality. The largest proportion of reviews addressed psychological and pharmacological interventions, followed by interdisciplinary, other (e.g., dietary), and physical interventions. Most common outcomes included pain, physical, emotional, and role functioning and quality of life. Treatment satisfaction and adverse events were less common, with minimal report of sleep or economic factors. Most patient-oriented research priorities had not been investigated.

**Conclusions**: Sufficient quality evidence is available to guide evidence-informed policies in pediatric chronic pain, most notably regarding psychological and pharmacological interventions. Numerous evidence gaps in patient-oriented research priorities and treatment outcomes should guide prioritization of research funds, as well as study aims and design.

## RÉSUMÉ

**Contexte**: Les études sur la douleur chronique chez les enfants se concentrent souvent sur une seule intervention ou sur une seule population, de sorte qu’il est difficile pour les responsables politiques et les décideurs de synthétiser rapidement les connaissances afin d'éclairer les priorités de financement et les politiques à plus grande échelle.

**Objectifs**: Les objectifs de cette étude étaient (1) de créer une carte présentant les lacunes dans les données probantes en ce qui concerne les interventions pour la douleur chronique chez les enfants et (2) de répertorier les lacunes dans les données probantes existantes par rapport aux priorités de recherche axées sur le patient récemment déterminées.

**Méthodes**: Nous avons effectué une revue systématique des revues systématiques évaluées par des pairs ou des guides de pratique clinique portant sur les interventions en matière de douleur chronique pédiatrique publiés en langue anglaise au cours des 20 dernières années. Des recherches ont été menées dans les bases de données Medline, Embase, PsycINFO, Web of Science, CINAHL et SCOPUS jusqu’au 3 juin 2019 inclusivement. La qualité des revues a été évaluée à l’aide d’AMSTAR-2.

**Résultats**: Sur 4168 résumés uniques, 50 revues systématiques (dont deux guides de pratique clinique) portant sur diverses populations pédiatriques souffrant de douleur chronique et divers milieux d'intervention ont été incluses. Un tiers d’entre elles ont été jugées de haute qualité, tandis que la moitié était jugée de basse à très basse qualité. La majeure partie des revues portait sur des interventions psychologiques et pharmacologiques, tandis que les autres portaient sur des interventions interdisciplinaires, d’autres types d’interventions (ex.: nutritionnelles) et des interventions physiques. Les issues les plus courantes comprenaient la douleur, le fonctionnement physique, émotionnel et de rôle, ainsi que la qualité de vie. La satisfaction à l'égard du traitement et les effets indésirables étaient moins souvent abordés, tandis que le sommeil et les facteurs économiques étaient peu mentionnés. La majeure partie des priorités de recherche axées sur les patients n'avait pas fait l’objet d’études.

**Conclusions**: Il existe suffisamment de données probantes de qualité pour guider les politiques fondées sur des données probantes en matière de douleur chronique pédiatrique, surtout en ce qui concerne les interventions psychologiques et pharmacologiques. L’existence de nombreuses lacunes dans les données probantes concernant les priorités de recherche axées sur le patient et les issues de traitement devrait guider la hiérarchisation du financement de la recherche, ainsi que les objectifs et la conception des études.

Chronic pain is a leading cause of disability and morbidity for children and adults.^1^ Despite this, chronic pain was only recently recognized as its own disease through the inclusion of new chronic pain diagnostic codes in the World Health Organization’s *International Classification of Diseases*, 11th revision, in 2018.^2^ The classification of chronic primary pain as a disease in its own right facilitates the progress of large-scale national policies to improve chronic pain management across the life span, many of which are underway in countries around the world.^3–5^ Furthermore, an evidence-based policy focus on improved chronic pain management is critical alongside policy efforts to address the opioid epidemic.^6^

The development of effective health policies is informed by both scientific evidence and stakeholder experience to ensure relevance and tailored implementation to the local context. Our group recently completed a national priority-setting partnership that engaged people with lived experience with pediatric chronic pain, family members, and treating health care providers across Canada to identify the top priorities for pediatric chronic pain research and care.^7^ The final top ten identified patient-oriented priorities direct the need for more evidence on prevention and treatment, as well as an improved understanding of the impact of pediatric chronic pain, delivery, access to care, and coordination of care. On its own, this priority-setting work provides a guided call to action for policymakers, decision makers, and researchers—from basic science to clinical research to health systems design—to address identified patient-oriented priorities; however, the uptake of these priorities may be limited by a lack of information about what research already exists in these areas. Current systematic reviews in pediatric chronic pain are often very niche and focus on one type of intervention.^8–10^ This makes it difficult for policymakers and decision makers to quickly synthesize knowledge across a variety of intervention modalities to inform clinical practice policy and research funding priorities. This is problematic given the current recommendation for multimodal care to thoroughly address biopsychosocial contributors to pediatric chronic pain.^11^

Although pediatric pain research is growing rapidly,^12^ there remains a disconnect between existing scientific evidence and current clinical practice, a further challenge to developing pediatric chronic pain policy.^13^ Estimates suggest that it can take up to 17 years for research to impact patient care,^14^ and many children and adolescents with chronic pain struggle to access evidence-based treatment.^15–17^ There is an identified need for more effectual and efficient knowledge mobilization in pediatric pain.^13^ The availability of high-quality evidence synthesis is a key step in the process of moving generated scientific knowledge into sustainable action, as outlined in the knowledge-to-action framework.^18^ Evidence and gap maps have emerged as an effective knowledge translation evidence synthesis tool to inform evidence-informed policymaking and the development of strategic research agendas.^19,20^ Like other evidence synthesis methods, such as a Cochrane reviews,^21^ evidence and gap maps are rigorous in their search for and assessment of research evidence^19,22^; however, they differ in their primary goal, which is to review the breadth and quality of available evidence compared to determining the efficacy of single specific interventions. This shift in focus facilitates the strategic identification of key gaps where little or no evidence exists or areas currently with only poor quality research.^19,22^ The use of schematic visual representation of findings in evidence and gap maps also makes research evidence more easily accessible and usable to researchers and decision makers.^19,22^

Our primary goal was to create a contemporary evidence and gap map of systematic reviews of all interventions for pediatric chronic pain. Our secondary aim was to identify gaps between existing evidence and recently identified patient-oriented research priorities for pediatric chronic pain.^7^ Given that many pediatric chronic pain interventions have limited evidence,^9,23^ we expected many priority areas to be lacking high-quality research evidence.

## Methods

### Protocol and Registration

This review adheres to PRISMA reporting guidelines for systematic reviews.^24^ A protocol was registered for this review in February 2018 on PROSPERO: CRD42018086817. The current review presents a minor modification from our original review protocol that outlines our initial intent to conduct a traditional overview of systematic reviews.^25^ The decision to modify the current review to an evidence and gap map was made following consultation with an international expert in evidence-informed health policy (Dr. John Lavis, personal communication, April 17, 2019) and in response to emerging national chronic pain policy efforts through the development of the Government of Canada’s Canadian Pain Task Force.^3^ It was felt that an evidence and gap map would better achieve our primary goal of uptake of evidence and patient priorities by policymakers and decision makers and identify clear gaps to guide research efforts in key areas identified by patients and clinicians as priorities.

Evidence and gap maps provide an overview of the availability and quality of evidence of a sector, in this case interventions for pediatric chronic pain.^19,20,22,26^ Recommended evidence and gap map methodology includes completion of six primary steps: development of scope, inclusion criteria, systematic review of the literature, data extraction, analysis, and visualization.^19,22,27^ Evidence and gap maps are underpinned by a rigor similar to that of other systematic review methodology.^28^ As such, reporting of the evidence and gap map methods in this article adhere to the PRISMA statement for reporting of systematic reviews and meta-analyses.^24^ The current review adheres to our original protocol with regards to the stated review question, search strategy, type of study, participants/population, interventions, risk of bias/quality assessment, narrative synthesis, and report by type of intervention modality. The current review diverges only from that outlined in our original protocol in that the current review now is restricted to publications within the past 20 years (since 1999), no longer extracts specific efficacy findings for intervention outcomes, and does not conduct subgroup analyses by type of chronic pain condition; additionally, the review now includes an evidence and gap map.

A completed evidence and gap map provides a simple and accessible visual summary of existing systematic review evidence for various types of interventions in pediatric chronic pain across selected outcome domains.^19,20,22,26^ The rows of the evidence and gap map list the types of interventions and the columns list the outcome domains. Each cell shows the number and quality of systematic reviews that contain evidence on that combination of intervention and outcome domain. In doing so, evidence and gap maps identify areas where little or no evidence exists (“absolute gaps”) and areas where there is systematic review evidence is available but is either out of date and/or of poor quality (“synthesis gaps”).^19,20,22,26^ Evidence and gap maps can be used to inform strategic research investment by highlighting intervention and outcome areas where new primary studies and/or systematic reviews can add value. They can also be used to inform decision making by capturing best available evidence that can then be compared against existing policy or programming to inform discussions about areas of prioritizing future research, policy, or investment.^19,20,22,26^

### Eligibility Criteria

Papers were eligible for inclusion if they

1. were peer-reviewed published systematic review or clinical practice guidelines;
2. were published in English;
3. included at least 50% or more reviewed studies focused on children or adolescents ≤18 years old or reported findings from pediatric studies separately;
4. included randomized and nonrandomized studies focused on any intervention for any type of chronic pain (defined as pain lasting at least 3 months or longer and/or pain described as “chronic,” “recurrent,” or “persistent”); and
5. reported on at least one primary or secondary outcome included in PedIMMPACT recommended for clinical trials in pediatric chronic pain (that is, pain intensity, physical functioning, emotional functioning, role functioning, quality of life, sleep, global treatment satisfaction, economic factors, and/or adverse events).^29^

Systematic reviews were excluded if they focused exclusively on chronic pain diagnosis or assessment and/or only reported prevalence. Prior iterations of eligible reviews were also excluded, as well as reviews/clinical practice guidelines published >20 years ago, given the availability of more current up-to-date evidence. Reviews including any type of intervention study design were included given recognized difficulty in conducting randomized controlled trials (RCTs) for some intervention modalities in pediatric populations (e.g., pharmacological) and given that interventions needed to address identified patient-oriented research priorities may not lend themselves easily to randomized study designs (e.g., school-based interventions).

Given variability in evidence synthesis methodology, requirements for being defined as a systematic review and/or clinical practice guideline were drawn from those used by the James Lind Alliance.^30^ Thus, a systematic review was defined as a review that attempts to identify, appraise, and synthesize all of the empirical evidence that meets prespecified eligibility criteria to answer a given research question. Therefore, a systematic review typically states/identifies a research question, provides search terms, searches multiple scientific databases, and reviews titles, abstracts, and full-text publications against some identified inclusion criteria. Clinical practice guidelines are clearly defined as such and include a systematic review to inform development of the guidelines. If multiple iterations or updates of the same systematic review or clinical practice guideline were found and identified as such, only the most recently published version of the systematic review or clinical practice guideline meeting the eligibility criteria was reviewed to reflect the most up-to-date evidence.

### Search Strategy and Conduct

Searches were conducted in Medline, Embase, PsycINFO, Web of Science, CINAHL, and SCOPUS from database inception to June 3, 2019. Database search strategies were developed in collaboration with a pediatric medical librarian and experts in pediatric chronic pain care and research. A sample comprehensive search strategy for Medline is available in Supplementary Online Material 1.

### Study Selection

Database search results were imported into Covidence^31^ for study selection. Initial abstract screening was conducted independently by two review authors (K.A.B. and T.D.A.), and full-text screening was independently performed by two review authors (C.O. and T.D.A.), with conflicts resolved by a third author (K.A.B.).

### Data Extraction and Quality Ratings

Data were independently extracted by two review authors (K.A.B. and T.D.A.). Extracted data items included review sponsorship, country, author, primary review objective, inclusion/exclusion criteria, date of literature search, inclusion of meta-analysis, types of reviewed studies (RCT or nonrandomized study [NRS]), total number of reviewed studies, number of reviewed studies focused on pediatric chronic pain intervention, population of reviewed studies (type of chronic pain/disease), setting of reviewed studies (e.g., outpatient, inpatient, emergency, etc.), type of intervention (pharmacological, psychological, physical, interdisciplinary, other), comparator groups, inclusion of quality of evidence rating, inclusion of PedIMMPACT^29^ recommended outcomes (pain intensity, physical functioning [e.g., mobility, disability], emotional functioning [e.g., anxiety, depression], role functioning [e.g., school attendance], quality of life, sleep, global treatment satisfaction, economic factors [e.g., cost, health care utilization, parent missed workdays], and/or adverse events), and time of outcome assessments.

When eligible systematic reviews included nonrelevant data (that is, pertaining to adults and/or nonchronic pain pediatric samples), only data relevant to reviewed studies focused on pediatric chronic pain were extracted. Supplementary material was accessed to inform data extraction and quality assessment if cited in published eligible systematic reviews. Separately reported systematic reviews that informed eligible clinical practice guidelines were accessed online (published and unpublished) and informed data extraction and quality assessment.

Risk of bias/quality of assessment of all eligible systematic reviews was conducted using the AMSTAR-2.^32^ The AMSTAR-2 critical appraisal tool includes 16 items that are rated to assess the quality of systematic reviews that include randomized or nonrandomized studies of health care interventions or both. Items address the review’s reporting of review criteria including elements of PICO (Population, Intervention, Comparator group, and Outcome), a *priori review protocol registration*, justification of study design selection, *adequacy of literature search*, study selection and data extraction in duplicate, *justification for excluding individual studies*, adequate description of included studies, *risk of bias from individual studies included in the review*, report of funding of included studies, *appropriateness of meta-analytical methods* (if applicable), *consideration of risk of bias when interpreting the review results*, and *assessment and likely impact of publication bias* (italics denote critical domains). Quality assessments for all eligible systematic reviews were rated independently by two authors (K.A.B. and C.O.), with disagreements resolved by consensus. Each systematic review was summarized by an single overall quality rating reflecting confidence in the results of review.^32^ Overall quality ratings are described as follows:

- **High**: No or one noncritical weakness; the systematic review provides an accurate and comprehensive summary of the results of the available studies that address the question of interest.
- **Moderate**: More than one noncritical weakness; the systematic review has more than one weakness but no critical flaws. It may provide an accurate summary of the results of the available studies that were included in the review.
- **Low**: One critical flaw with or without noncritical weaknesses; the systematic review has a critical flaw and may not provide an accurate and comprehensive summary of the available studies that address the question of interest.
- **Critically Low**: More than one critical flaw with or without noncritical weaknesses; the systematic review has more than one critical flaw and should not be relied on to provide an accurate and comprehensive summary of the available studies.

Eligible systematic reviews and clinical practice guidelines were also independently coded by two authors (K.A.B. and C.O.) for relevance to each of the top ten patient-oriented research priorities for pediatric chronic pain identified in the Partnering For Pain priority-setting partnership.^7^ In brief, Partnering For Pain engaged hundreds of diverse Canadians with lived experience with pediatric chronic pain, family members, and multidisciplinary health care providers across four priority setting phases using the James Lind Alliance Priority Setting Partnership methodology. The James Lind Alliance methodology is recognized as being robust, strategic, objectively based and inclusive, and promoting equity in patient voices.^33^ In phase 1, 215 Canadians (86 patients [40.0%], 56 family members [26.0%], and 73 health care providers [34.0%]) submitted 540 potential priorities that were developed into 112 unique research questions (phase 2). Of the 112 questions, 63 were rated for importance by 57 participants (19 patients [33%], 17 family members [30%], and 21 health care providers [37%]) in phase 3. In phase 4, 20 participants (6 patients [30%], 6 family members [30%], and 8 health care providers [40%]) discussed the 25 most highly rated questions and reached consensus on the final top ten.^7^ The participant group was diverse with regards to age, sex, ethnicity, geographic location, chronic pain condition, care setting, and health care profession. A thorough discussion about the rationale, methodology, findings, and limitations of the Partnering For Pain priority-setting partnership is available in our previous peer-reviewed publication.^7^

## Results

### Study Selection

**Figure 1.**
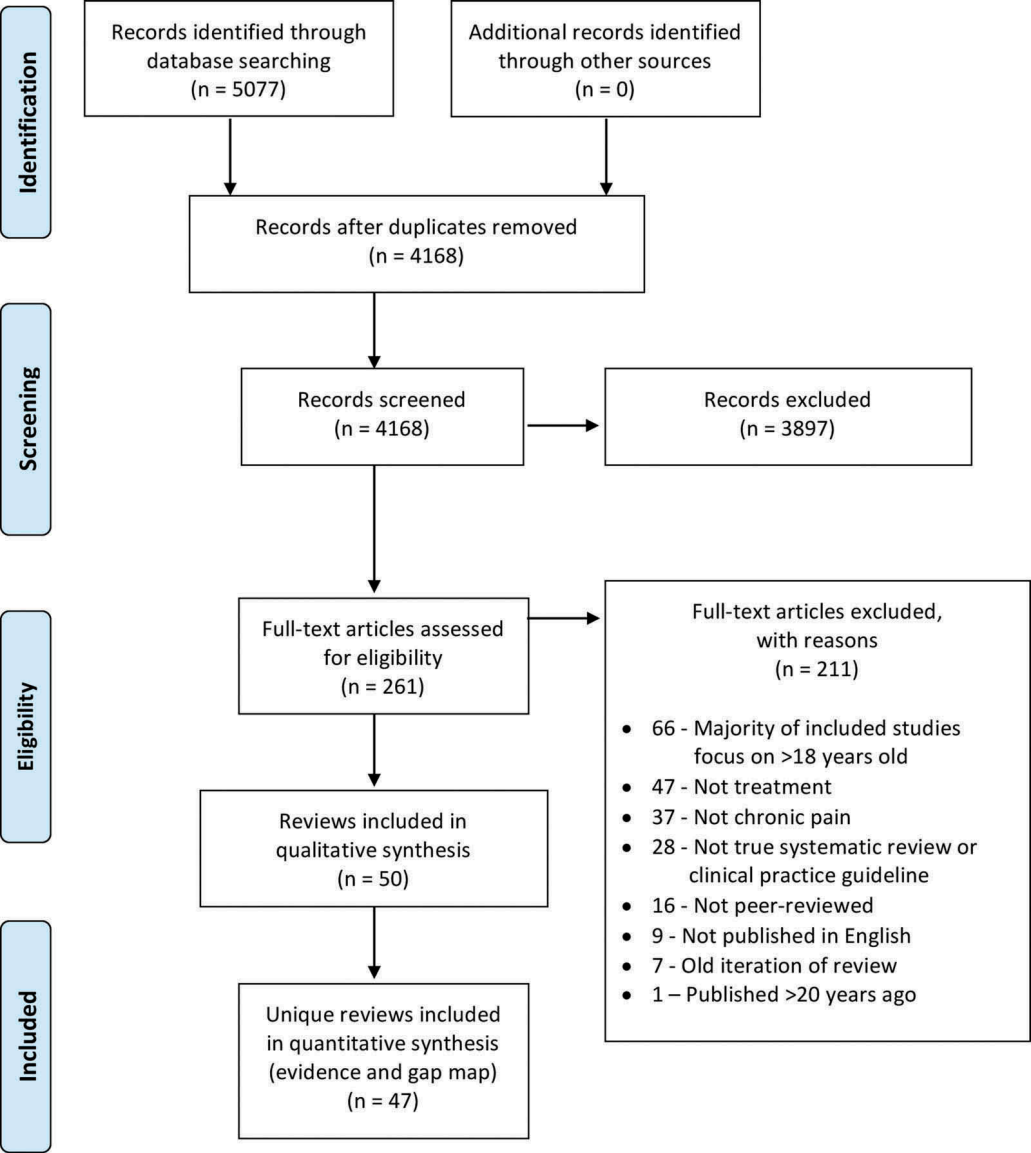
PRISMA review flowchart

Database searches identified 5077 records. After duplicates were removed, 4168 unique abstracts remained for review. Of these, 3897 were deemed not eligible. A total of 261 full texts were reviewed and 211 were excluded. Fifty full texts met inclusion criteria. See Figure 1 for the PRISMA review flowchart, including reasons for full-text exclusion.

### Study Characteristics

Of the 50 full texts meeting review inclusion criteria, 2 reported a related systematic review and clinical practice guideline for pediatric chronic abdominal pain^34,35^ and 2 reported a related systematic review and clinical practice guideline for pediatric chronic widespread pain.^36,37^ Data extraction and quality assessment were combined for each pair of related systematic reviews and clinical practice guidelines. One additional paper^38^ was a summary of 3 other included systematic reviews.^10,39,40^ Data extraction and quality ratings were not conducted for the summary review because information was obtained from the original included systematic reviews. Thus, data and quality ratings are reported for 47 unique reviews/clinical practice guidelines. Of these, 24 (51.6%) included meta-analyses. Almost half of the reviews reported no funding or sponsorship (*n = *22; 46.8%). See Tables 1 and 2 for characteristics and outcomes for each included review.

**Table 1.** Characteristics of included reviews

Author, year	Aim	Meta-analysis	Search date	No. and type of relevant studies	Age and chronic pain population(s)	Setting(s)	Type of intervention(s)	AMSTAR-2 quality rating
Abbott et al., 2017^39^	To determine the effectiveness of psychosocial interventions for reducing pain in school-aged children with recurrent abdominal pain	Yes*	06/2017	18 RCTs	6–18 years Recurrent abdominal pain, functional abdominal pain, IBS	Gastroenterology and pain outpatient clinic, community	Psychological	High
Abujaradeh et al., 2018^41^	To determine the benefits/efficacy of mindfulness-based interventions implemented among adolescents with chronic diseases in clinical settings	No	11/2017	8 RCTs and NRS	12–18 years Mixed chronic pain	Tertiary care multidisciplinary pain clinic or hospital setting	Psychological	Low
American Academy of Pediatrics, 2005^34^ and Di Lorenzo et al., 2005^35^	To examine the diagnostic and therapeutic value of a medical and psychologic history, diagnostic tests, and pharmacological and behavioral therapy for chronic abdominal pain	No + CPG	??/2002	8 RCTs and NRS	5–17 years Abdominal pain	??	Psychological Pharmacological Other	Low
Anie and Green, 2015^74^	To examine the evidence that psychological interventions improve the ability of people with sickle cell disease to cope with their condition	Yes*	02/2015	4 RCTs	6–18 years+ Sickle cell disease	“Urban setting”	Psychological	Moderate
Arruda et al., 2018^62^	To review the comorbidities and treatment of chronic migraine in children and adolescents in the last ten years, summarizing evidence-based recommendations for disease management	No	??/2017	4 RCTs and NRS	7–17 years Headaches, migraine	Inpatient, outpatient headache clinics	Pharmacological Interdisciplinary	Critically low
Badawy et al., 2018^75^	To (1) review the types of technological tools used for self-management of sickle cell disease, (2) discover and describe what self-management activities these tools were used for, and (3) assess the efficacy of these technologies in self-management	No	11/2016	5 RCTs and NRS	13–18 years+ Sickle cell disease	e-Health	Psychological Interdisciplinary	Low
Bailey and McManus, 2008^63^	To evaluate which treatment could be effective in the emergency department for children with migraine and status migrainous	No	06/2007	14 RCTs	4–18 years Migraines	Emergency department, outpatient neurology clinics	Pharmacological	Critically low
Barnes, 2015^64^	To examine treatments for acute attacks and the effects of pharmacological prophylaxis for migraine headache in children	No	06/2014	23 RCTs and reviews of RCTs	3–18 years Migraines, headaches	??	Pharmacological	Moderate
Bredlau et al., 2013^71^	To review the use of ketamine in the treatment of cancer-related pain in adults and children	No	02/2013	5 NRS	4–19 years Cancer pain	??	Pharmacological	Low
Brent et al., 2009^59^	To evaluate behavioral and psychological treatments applied to pediatric functional gastrointestinal disorders	No	??/2007	12 RCTs and NRS	4–18 years Recurrent abdominal pain, IBS, functional abdominal pain, functional dyspepsia, stomachache, stomach pain	Outpatient, community	Psychological Other Interdisciplinary	Critically low
Cairns et al., 2018^76^	To evaluate interventions for patellar tendon–related pain in children and adolescents	No	11/2017	3 RCTs and NRS	10–18 years Patellar tendon pain, Osgood-Schlatter’s	University physical therapy, secondary care orthopedics, orthopedic surgery department	Physical Pharmacological Interdisciplinary	Moderate
Cohen et al., 2017^67^	To assess the effects of psychosocial therapies on pain and function in children with rheumatic diseases	No	09/2015	5 RCTs and NRS	5–18 years Juvenile fibromyalgia, juvenile arthritis	??	Psychological	Low
Cooper, Wiffen, et al., 2017^42^	To assess the analgesic efficacy of antiepileptic drugs used to treat chronic non-cancer pain in children and adolescents	Yes*	09/2016	2 RCTs	7–18 years Fibromyalgia, CRPS-I or neuropathic pain	Tertiary care centers	Pharmacological	High
Cooper, Heathcote, et al, 2017^72^	To assess the analgesic efficacy of nonsteroidal anti-inflammatory drugs used to treat cancer-related pain in children and adolescents	Yes*	02/2017	0 RCTs	<18 years Cancer-related pain		Pharmacological	High
Cooper, Fisher, et al., 2017^8^	To assess the analgesic efficacy of opioids used to treat chronic non-cancer pain in children and adolescents	Yes*	09/2016	0 RCTs	<18 years Chronic non-cancer pain		Pharmacological	High
Eccleston et al., 2015^43^	To evaluate the efficacy of psychological therapies that include parents of children and adolescents with chronic illnesses	Yes*	07/2014	14 RCTs	<19 years Headache, sickle cell, recurrent abdominal pain, IBS, fibromyalgia, arthritis, mixed pain conditions	Outpatient clinics, community	Psychological	High
Eccleston et al., 2017^44^	To assess the analgesic efficacy of nonsteroidal anti-inflammatory drugs used to treat chronic non-cancer pain in children and adolescents	Yes*	09/2016	7 RCTs	2–18 years Chronic non-cancer pain	Pediatric rheumatology tertiary care units, pediatric centers, clinical centers	Pharmacological	High
Egunsola et al., 2019^45^	To examine the efficacy and safety of gabapentin and pregabalin for pain in children and adolescents	No	11/2017	2 RCTs	7–18 years CRPS, neuropathic pain, fibromyalgia	??	Pharmacological	High
Fellas et al., 2017^70^	To review the evidence for physical and mechanical interventions for lower-limb problems in juvenile idiopathic arthritis	Yes	06/2015	2 RCTs	5–19 years Juvenile idiopathic arthritis	Hospitals, pediatric rheumatology clinics	Physical	High
Ferro and Speechley, 2008^68^	To review of the prevalence of complementary and alternative medicine use in juvenile idiopathic arthritis, determinants of use, and outcomes associated with various therapies	No	10/2008	3 RCTs and NRS	“Children” Juvenile idiopathic arthritis	??	Psychological Other	Critically low
Fisher et al., 2018^46^	To determine any effect of psychological therapy for chronic and recurrent pain in children and adolescents	Yes*	05/2018	47 RCTs	6–18 years Mixed chronic pain, headaches, abdominal pain, IBS, inflammatory bowel disease, fibromyalgia, temporomandibular disorders, sickle cell disease	Hospitals, outpatient clinics, patient home	Psychological	High
Fisher et al., 2019^47^	To determine the efficacy of psychological therapies delivered remotely for the management of chronic pain in children and adolescents	Yes*	05/2018	10 RCTs	7–17 years Headache, juvenile idiopathic arthritis, abdominal pain, musculoskeletal, sickle cell disease	Via technology at home (Internet, CD-ROM)	Psychological	High
Hechler et al., 2015^48^	To review studies the effects of pediatric intensive interdisciplinary pain treatment	Yes	02/2014	10 RCTs and NRS	8–22 years Mixed (CRPS, headache, neuropathic, idiopathic, fibromyalgia/diffuse, disease-related, back, abdominal, pelvic, musculoskeletal, etc.)	Inpatient, day hospital setting, rehabilitation	Interdisciplinary	Moderate
Horvath et al., 2012^60^	To evaluate the effect of dietary fibers for treating abdominal pain-related functional gastrointestinal disorders in children	No	12/2011	3 RCTs	3–16 years Abdominal pain	Hospitals, community clinics	Other	Low
Huertas-Ceballos 2008^77^^,^	To determine the effectiveness of medication for recurrent abdominal pain in school-age children	Yes*	12/2006	3 RCTs	5–18 years Abdominal migraine, recurrent abdominal pain, dyspepsia, IBS	??	Pharmacological Other	High
Huertas-Ceballos 2008^78^	To determine the effectiveness of psychosocial interventions for recurrent abdominal pain or IBS in school-age children	Yes*	12/2006	6 RCTs	5–18 years Recurrent abdominal pain	Community, outpatient clinic	Psychological Other Interdisciplinary	High
Kichline and Cushing, 2019^49^	To evaluate the effect of exercise on pediatric chronic pain	Yes	12/2016	11 RCTs and NRS	6–16 years Arthritis, fibromyalgia, low back pain, cancer, mixed	??	Physical	Low
Liossi et al., 2019^50^	To review the effectiveness of interdisciplinary interventions in the management of pediatric chronic pain	Yes	03/2018	28 RCTs and NRS	6–21 years Mixed (headache, abdominal pain, back, migraines, CRPS, pelvic, neuropathic, widespread/fibromyalgia, musculoskeletal), oncology (tumors, leukemia), arthritis	Inpatient, day treatment, outpatient	Interdisciplinary	High
Lonergan, 2016^51^	To examine the effectiveness of cognitive behavioral therapy in the treatment of chronic pain in children and adolescents	Yes	12/2014	9 RCTs	6–18 years Recurrent abdominal pain, fibromyalgia, headache or migraine	Outpatient, university, patient home	Psychological	Critically low
Martin et al., 2017^40^	To review the effectiveness of pharmacological interventions for recurrent abdominal pain in school-age children	Yes*	06/2016	16 RCTs	5–18 years Recurrent abdominal pain	Hospital pediatric outpatient clinics	Pharmacological	High
Michel et al., 2011^52^	To review relevant pediatric buprenorphine data, particularly in children suffering chronic pain	No	??	12 NRS	“Children” Oncology, postoperative		Pharmacological	Critically low
Newlove-Delgado et al., 2017^10^	To examine the effectiveness of dietary interventions in improving pain in school-age children with recurrent abdominal pain	Yes*	06/2016	19 RCTs	4–18 years Recurrent abdominal pain, functional gastrointestinal disorders, IBS	Pediatric gastroenterology clinics, primary care pediatric practices, community clinics	Other	High
Ng et al., 2017^65^	To review the efficacy of cognitive–behavioral therapy for pediatric migraine	Yes	05/2016	17 RCTs	7–18 years Migraine	Medical setting, school	Psychological	Critically low
Nijhof et al., 2018^69^	To review the evidence for nonpharmacological treatment for chronic musculoskeletal pain in pediatric rheumatic disease	No	10/2017	11 RCTs and NRS	5–18 years Juvenile idiopathic arthritis, systemic lupus erythematosus	??	Psychological Physical Interdisciplinary	Low
Ostojic et al., 2018^80^	To determine the efficacy of interventions for the management of pain in children and adolescents with cerebral palsy	No	04/2018	50 RCTs and NRS	<18 years Cerebral palsy (postoperative, hypertonia, cerebral palsy spastic hip disease)	Hospital, outpatient clinic	Physical Pharmacological Other Interdisciplinary	Low
Palermo et al., 2010^53^	To examine the efficacy of psychological interventions for pain and emotional and physical functioning in children	Yes	08/2008	18 RCTs	4–18 years Headache, migraine, abdominal pain, fibromyalgia	Hospital or clinic	Psychological	Moderate
Scheper et al., 2013^81^	To review state of the art of diagnostics and treatment of generalized joint hyper mobility and joint hyper mobility syndrome in children and young adults	No	??	3 RCTs	0–18 years Osteogenesis imperfecta, generalize joint hyper mobility, joint hyper mobility syndrome, Ehlers-Danlos	??	Physical Other	Moderate
Shah et al., 2016^54^	This review the role of interventional procedures in the treatment of chronic pain in children and adolescents	No	03/2013	133 NRS	“Children” Migraine, headache, neuropathic, postoperative, cystic fibrosis, sickle cell, juvenile idiopathic arthritis, head, abdomen, fibromyalgia, CRPS, phantom limb, back, chest, cancer	Inpatient, outpatient settings	Pharmacological	Critically low
Sprenger et al., 2011^61^	To examine the effectiveness of psychological therapies for children with recurrent abdominal pain	Yes	11/2009	10 NRS	4–18 years Recurrent abdominal pain	??	Psychological	Critically low
Tomé-Pires and Miró, 2012^55^	To review hypnotic treatments for chronic and cancer procedure-related pain in children	No	05/2010	2 RCTs	6–18 years Headache, abdominal pain	??	Psychological	Critically low
Trautmann et al., 2006^66^	To describe the state of evidence in the treatment of pediatric headaches	Yes	07/2004	23 RCTs	7–18 years Headache	Outpatient clinic, school	Psychological	Low
Velleman et al., 2010^56^	To review the use of computerized cognitive behavioral therapy with children and adolescents with pain	Yes	??/2008	4 RCTs	7–17 years Headaches, recurrent abdominal pain, musculoskeletal	Via computer in outpatient clinic, community	Psychological	Low
Weydert et al., 2003^57^	To review treatments for recurrent abdominal pain in children	No	??/2001	10 RCTs	3–18 years Recurrent abdominal pain, IBS, abdominal migraine	Primary and tertiary care, community	Psychological Pharmacological Other Interdisciplinary	Critically low
Wicksell et al., 2015^58^	To provide an overview of research on acceptance and commitment therapy for youths with physical concerns	No	??	8 RCTs and NRS	7–18 years Chronic pain	??	Psychological	Critically low
Wiffen et al., 2017^73^	To assess the analgesic efficacy, of opioids used to treat cancer-related pain in children and adolescents	Yes*	02/2017	0 RCTs	<18 years Cancer-related pain	—	Pharmacological	High
Yeung et al., 2017^79^	To review characteristics and management of endometriosis in adolescents	No	??	8 NRS	10–25 years Endometriosis	??	Other Interdisciplinary	Critically low
Zernikow et al., 2012^37^ and Häuser et al., 2012^36^	To provide a definition, diagnosis, and therapy of chronic widespread pain and so-called fibromyalgia syndrome in children and adolescents	No + CPG	12/2010	?? RCTs and NRS	“Children and adolescents” Chronic widespread pain, fibromyalgia	Inpatient, outpatient	Psychological Physical Pharmacological Interdisciplinary	Moderate

* = Cochrane review; RCT = randomized controlled trial; IBS = irritable bowel syndrome; NRS = nonrandomized study; CPG = clinical practice guideline; ?? = unclear/unknown; CRPS = complex regional pain syndrome; — = no studies.

**Table 2.** Interventions and outcomes of included reviews

			PedIMMPACT outcomes								
Author, year	Intervention(s)	Longest follow-up time	Pain	Physical functioning	Emotional functioning	Role functioning	Quality of life	Sleep	Economic factors	Treatment satisfaction	Adverse events
Abbott et al., 2017^39^	CBT, hypnosis, written self-disclosure, yoga	>1 year	✔	✔	✔	✔	✔				
Abujaradeh et al., 2018^41^	mindfulness-based interventions	6 months	✔	✔	✔	✔	✔				
American Academy of Pediatrics, 2005^34^ and Di Lorenzo et al., 2005^35^	CBT, coping skills, famotidine, pizotifen, peppermint oil, fiber supplement, lactose-free diet, surgery	3 years	✔			✔				✔	✔
Anie and Green, 2015^74^	Education, various psychotherapies, art therapy	1 year	✔		✔	✔	✔		✔		
Arruda et al., 2018^62^	Amitriptyline, corticoid infusion, peripheral nerve blocks. onabotulinumtoxina injections, pharmacological + CBT	1 year	✔	✔							
Badawy et al., 2018^75^	Internet-delivered CBT, psychoeducation	7 months	✔	✔	✔			✔	✔	✔	
Bailey and McManus, 2008^63^	Acetaminophen, ibuprofen, zolmitriptan, sumatriptan, rizatriptan, dihydroergotamine, ketorolac, prochlorperazine	24 hours	✔								✔
Barnes, 2015^64^	Almotriptan, ibuprofen, topiramate, flunarizine, sumatriptan, rizatriptan, zolmitriptan, eletriptan, propanolol, flunarizine	4 months	✔	✔							✔
Bredlau et al., 2013^71^	Ketamine	75 days	✔								✔
Brent et al., 2009^59^	Relaxation, hypnotherapy, CBT, psychoeducation, dietary (fiber), psychological + diet	1 year	✔	✔	✔	✔					
Cairns et al., 2018^76^	Shockwave treatment + light activity, other physical exercises, local anesthetic, surgery + physical therapy	2–3 years	✔	✔							✔
Cohen et al., 2017^67^	CBT, coping skills, relaxation, education, distraction	4 months	✔	✔			✔				✔
Cooper, Wiffen, et al., 2017^42^	Antiepileptics (pregabalin, gabapentin)	15 weeks	✔	✔		✔	✔	✔		✔	✔
Cooper, Heathcote, et al., 2017^72^	NSAIDs	—	—	—	—		—	—	—	—	—
Cooper, Fisher, et al., 2017^8^	Opioids	—	—	—	—		—	—	—	—	—
Eccleston et al., 2015^43^	CBT, behavioral intervention	1 year	✔	✔	✔	✔	✔		✔	✔	✔
Eccleston et al., 2017^44^	NSAIDs	1 year	✔							✔	✔
Egunsola et al., 2019^45^	Antiepileptics (pregabalin, gabapentin)	15 weeks	✔					✔			✔
Fellas et al., 2017^70^	Foot orthotics, neoprene inserts	6 months	✔	✔			✔				
Ferro and Speechley, 2008^68^	Relaxation, herbal therapy (*Tripterygium wilfordii*), massage therapy	6 months	✔	✔							
Fisher et al., 2018^46^	CBT, relaxation with or without biofeedback, coping skills, problem-solving therapy, intensive inpatient rehabilitation	1 year	✔	✔	✔	✔	✔	✔	✔	✔	✔
Fisher et al., 2019^47^	CBT (CD-ROM, Internet)	1 year	✔	✔	✔	✔	✔	✔		✔	✔
Hechler et al., 2015^48^	Variations of combined pharmacological, psychological, physical	2 years	✔	✔	✔	✔					
Horvath et al., 2012^60^	Dietary fiber	??	✔	✔		✔				✔	✔
Huertas-Ceballos 2008^77^	Pizotifen, famotidine, peppermint oil capsules	??	✔								
Huertas-Ceballos 2008^78^	CBT, family therapy, dietary fiber, psychological + fiber	1 year	✔	✔		✔	✔			✔	
Kichline and Cushing, 2019^49^	Aerobic exercise	6 months	✔								
Liossi et al., 2019^50^	Variations of combined pharmacological, psychological, physical, and other	2 years	✔	✔	✔	✔	✔	✔	✔		
Lonergan, 2016^51^	CBT, family therapy	1 year	✔	✔	✔						✔
Martin et al., 2017^40^	TCAs, antibiotics, 5-HT4 receptor agonists, antispasmodics, antihistamines, H2 receptor antagonists, serotonin antagonists, SSRIs, dopamine receptor antagonist	4 months	✔		✔	✔	✔	✔		✔	✔
Michel et al., 2011^52^	Buprenorphine	??	✔		✔						✔
Newlove-Delgado et al., 2017^10^	Probiotic-based interventions, fiber-based interventions, low FODMAP diets, fructose-restricted diet	4 months	✔	✔	✔	✔	✔				✔
Ng et al., 2017^65^	CBT, relaxation, biofeedback	1 year	✔	✔	✔	✔	✔				
Nijhof et al., 2018^69^	CBT, relaxation, biofeedback, education, physical conditioning, Pilates, resistive underwater exercises, physical therapy + biofeedback	6 months	✔	✔			✔				
Ostojic et al., 2018^80^	Physiotherapy, massage, TENS, intrathecal baclofen, botulinum toxin A, cyclic intravenous administration of pamidronate, fentanyl, clonidine, indomethacin, intrathecal morphine, amitriptyline, gabapentin, bupivacaine, ketamine, blocks/epidural, butorphanol, magnesium sulfate, laser therapy, surgery, physiotherapy + pharmacological	2 years	✔	✔	✔		✔			✔	
Palermo et al., 2010^53^	CBT, relaxation, biofeedback	1 year	✔		✔	✔	✔				
Scheper et al., 2013^81^	Physical training, Bobath treatment	18 months	✔	✔			✔				
Shah et al., 2016^54^	Neuroaxial blocks, peripheral blocks, sympathetic blocks, Bier blocks, neurostimulation, intrathecal baclofen, intra-articular steroids	??	✔								✔
Sprenger et al., 2011^61^	CBT, family-based, relaxation, biofeedback, yoga	??	✔								✔
Tomé-Pires and Miró, 2012^55^	Hypnosis	1 year	✔								
Trautmann et al., 2006^66^	CBT, biofeedback, relaxation, stress management	1 year	✔								
Velleman et al., 2010^56^	CBT (CD-ROM, Internet)	6 months	✔	✔		✔	✔			✔	
Weydert et al., 2003^57^	CBT, famotidine, pizotifen. botanicals (peppermint oil), diet (fiber, lactose avoidance), CBT + biofeedback + fiber	??	✔		✔	✔				✔	
Wicksell et al., 2015^58^	Acceptance and commitment therapy	3 months	✔	✔	✔						
Wiffen et al., 2017^73^	Opioids	—	—	—	—	—	—	—	—	—	—
Yeung et al., 2017^79^	NSAIDs, TCAs, SSRIs, trazodone or amitriptyline, cyclobenzaprine, laparoscopy, laparoscopic excision or treatment, surgical ablation or excision, surgery + postoperative hormones	??	✔				✔				
Zernikow et al., 2012^37^ and Häuser et al., 2012^36^	CBT, trauma therapy, family therapy, other psychotherapy, aerobic exercises, qi-gong, psychological + physical therapy	5+ years	✔	✔	✔	✔	✔	✔			✔

CBT = cognitive–behavioral therapy; — = no studies; NSAID = nonsteroidal anti-inflammatory drug; ?? = unclear/unknown; TCA = tricyclic antidepressants; SSRI = selective serotonin re-uptake inhibitor; FODMAP = fermentable oligosaccharides, disaccharides, monosaccharides and polyols; - = no studies.

#### Types of Populations

Most reviews (*n* = 19; 40.4%) included variations of mixed chronic pain populations (e.g., abdominal pain, headaches or migraines, widespread pain/fibromyalgia, complex regional pain syndrome, neuropathic pain, sickle cell disease, cancer pain, back pain, and/or pelvic pain).^8,37,41–58^ Reviews focused on single populations most frequently examined abdominal pain (*n* = 10; 21.3%),^10,35,39,40,57,59–61,77,78^ headaches or migraines (*n* = 5; 10.6%),^62–66^ rheumatological conditions (e.g., juvenile idiopathic arthritis, lupus; *n* = 4; 8.5%),^67–70^ cancer-related pain (*n* = 3; 6.4%),^71–73^ or sickle cell disease (*n* = 2; 4.3%).^74,75^ Single reviews focused on patellar tendon pain/Osgood-Schlatter’s (*n* = 1; 2.1%),^76^ cerebral palsy (*n* = 1; 2.1%),^80^ endometriosis (*n* = 1; 2.1%),^79^ or joint hypermobility/Ehlers-Danlos/osteogenesis imperfecta (*n* = 1; 2.1%).^81^

Reviews included children 2–18 years old. Most reviews included studies crossing childhood and adolescence (*n* = 45; 95.7%), with two reviews (4.3%) focused on adolescents (>12–18 years old).^41,75^ Five reviews also included studies with adults (>18 years old).^48,50,70,74,75^

#### Types of Settings

Reviews included studies conducted in a variety of settings, including primarily tertiary care or hospital settings (inpatient, day treatment, outpatient clinics, and emergency departments), followed by primary care or community-based clinics and, rarely, schools. Three reviews (6.4%) focused exclusively on “e-health” or remotely delivered interventions.^47,56,75^ The setting was not clearly reported in 12 (25.5%) reviews.

#### Types of Studies Included

The majority of reviews exclusively included RCTs or reviews of RCTs (*n* = 26; 55.3%). The remaining reviews included a variety of study designs, including nonrandomized intervention studies, cohort or observational studies, retrospective chart reviews, and case studies or case series (*n* = 21; 44.6%). Most reviews included at least one study with a comparator group (*n* = 41; 87.2%). Comparator groups included usual/standard medical care, waitlist controls, placebo or sham interventions, or other active interventions.

#### Types of Interventions

Though some reviews focused on singular types of intervention, others focused on varied types of treatment for a particular pain population or setting. Almost half of the reviews examined psychological interventions (*n* = 23; 48.9%), with 19 (40.4%) reviewing pharmacological interventions, 12 (25.5%) reviewing interdisciplinary interventions, 11 (23.4%) reviewing “other” interventions, and 7 (14.9%) reviewing physical interventions. The “other” types of treatments reviewed were primarily dietary (e.g., fiber, lactose avoidance), botanicals (e.g., peppermint oil, herbal therapy), and surgical interventions.

#### Types of Outcomes

Three reviews of pharmacological interventions found no eligible studies for inclusion^8,72,73^; as such, extraction of assessed outcomes was not possible for those reviews. Of the remaining 44 reviews, all (100%) reported on pain intensity, 27 (61.3%) reported on physical functioning, 20 (45.5%) reported on emotional functioning, 20 (45.5%) reported on role functioning, 21 (47.7%) reported on quality of life, 8 (18.2%) reported on sleep, 5 (11.4%) reported on economic factors, 13 (29.5%) reported on treatment satisfaction, and 20 (45.5%) reported on adverse events. Time points for outcome reporting ranged from immediately postintervention to hours, days, weeks, months, or up to 5 or more years later. Most reviews included some sort of risk of bias or quality ratings of included studies (*n* = 32; 72.7%).

### Quality of Systematic Reviews

**Figure 2.**
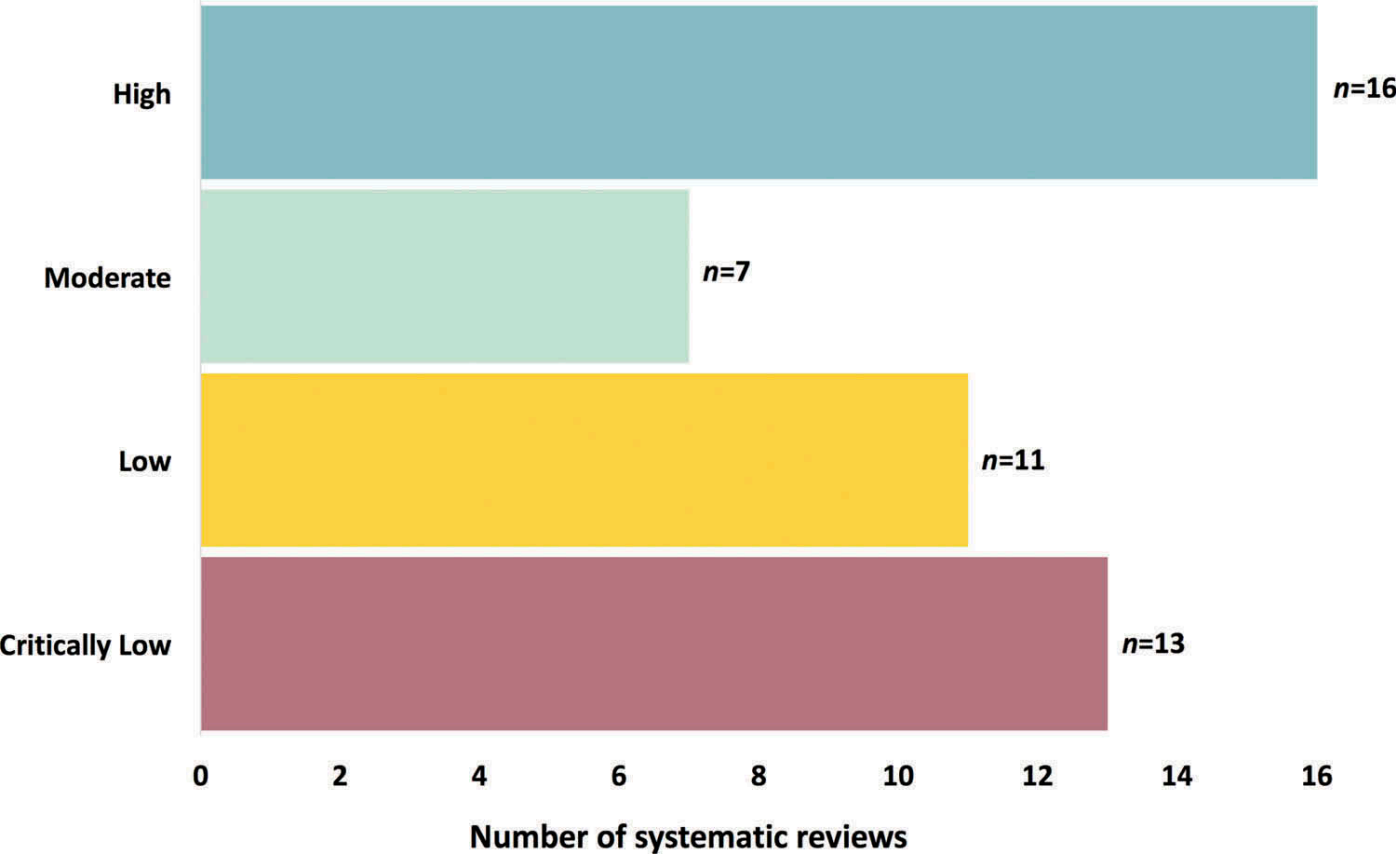
AMSTAR-2 quality ratings of included systematic reviews

See Figure 2 for a summary of the AMSTAR-2 quality ratings for the included reviews. Of the 47 reviews, the greatest number were rated as high quality (*n* = 16; 34.0%), followed by critically low quality (*n* = 13; 27.7%) and low quality (*n* = 11; 23.4%), with the fewest rated as moderate quality (*n* = 7; 14.9%). Reviews were primarily downgraded in quality for failing to register a review protocol or demonstrate clear evidence of review methods established a priori or failing to provide a list of excluded studies with justification, with fewer studies failing to use a comprehensive literature strategy, failing to include a satisfactory technique for assessing risk of bias, or failing to account for risk of bias in the interpretation of review results.

### Synthesis of Results

**Figure 3.**
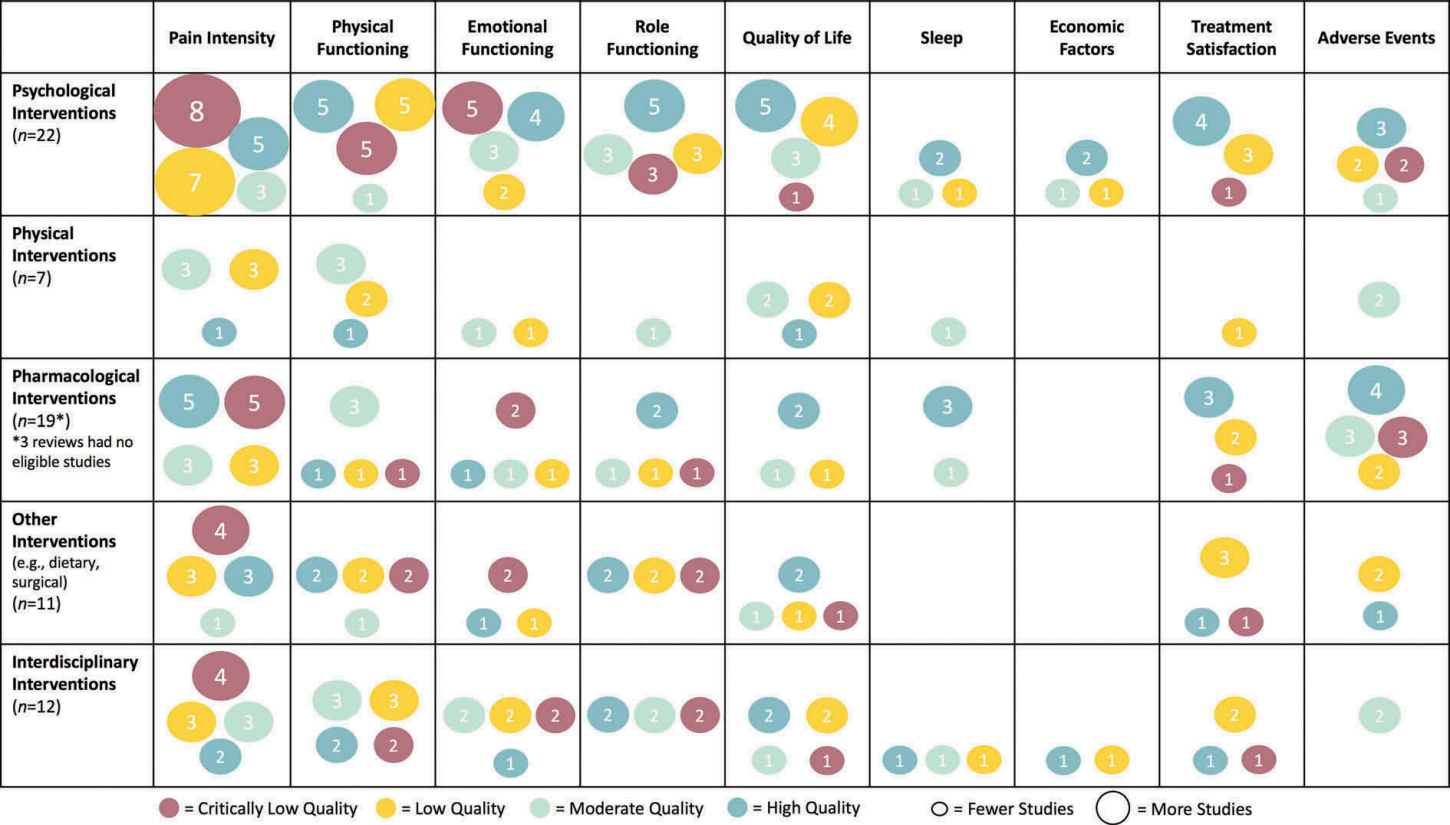
Evidence and gap map of interventions for pediatric chronic pain. The figure rows list the types of interventions and the columns list the PedIMMPACT outcome domains. Each cell shows the number and quality of included systematic reviews as assessed using AMSTAR-2 that contain evidence on that combination of type of intervention for pediatric chronic pain and outcome domain

See Figure 3 for the evidence and gap map summarizing the quality and number of included reviews relevant to each extracted treatment outcome of interest.

### Additional Analyses: Mapping to the Top Ten Patient-Oriented Research Priorities

**Figure 4.**
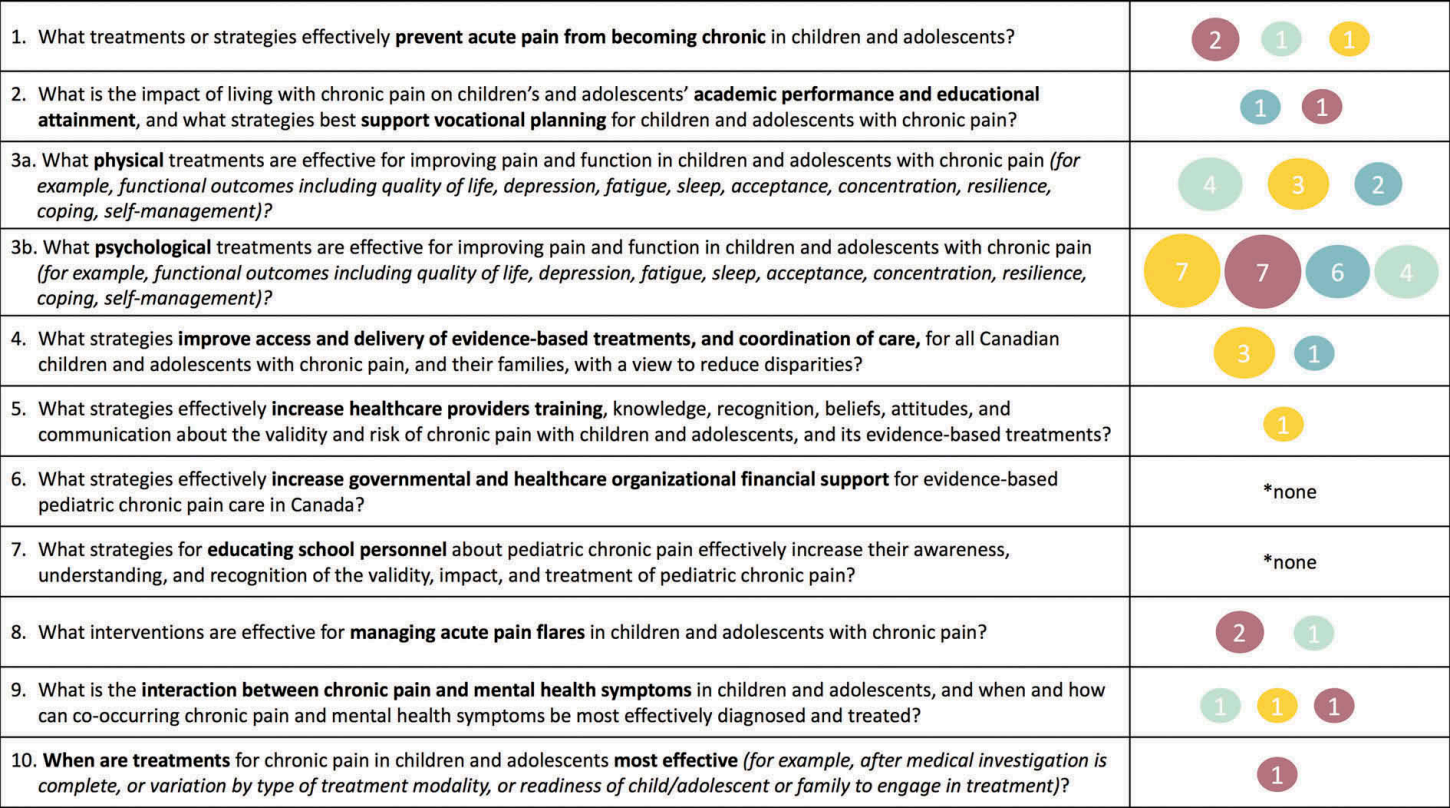
Summary of evidence for top ten patient-oriented research priorities in pediatric chronic pain

See Figure 4 for a summary of the quality and number of included reviews relevant to each of the top ten patient-oriented research priorities for pediatric chronic pain. All but two priorities had at least one relevant review and/or clinical practice guideline. Priority 3 (physical and psychological interventions) had the greatest number of relevant reviews (*n* = 9; 19.1% and *n* = 24; 51.1%, respectively), albeit primarily from reviews of low and critically low quality. Priority 1 (prevention of chronic pain) and priority 4 (improved access and delivery) were addressed by four reviews each (8.5%), and priority 2 (impact on education and vocational planning), priority 8 (managing acute pain flares), and priority 9 (treatment of co-occurring mental health symptoms) were addressed by only two to three reviews each (4.3–6.4%). Priority 5 (increase health care providers’ knowledge) and priority 10 (timing of interventions) had only one relevant review each (2.1%), and priority 6 (increase government and organization financial support) and priority 7 (educating school personnel) had no relevant reviews. Almost one third of included reviews and clinical practice guidelines did not address any of the patient-oriented research priorities (*n* = 15; 31.9%).

## Discussion

### Summary of Evidence

This systematic review offers a rigorous synthesis of available systematic reviews and clinical practice guidelines of interventions of any modality for pediatric chronic pain. The resulting evidence and gap map offers a succinct but thorough data visualization to effectively convey the current state of the evidence for use by key stakeholders, including members of the public, policymakers and decision makers, health care providers, and researchers alike. The broad scope of this review across intervention modalities and pediatric chronic pain populations, as well as its evidence and gap map methodology, uniquely positions its findings to be quickly and easily utilized.

This review reveals much about the contemporary state of synthesized evidence of interventions for pediatric chronic pain. It is promising for policymakers that many high-quality reviews exist to guide decisions (most of which were Cochrane reviews); however, more than half (55%) of included reviews were rated to be of low or critically low quality. It was surprising that only two clinical practice guidelines were identified. Most systematic reviews examine psychological interventions only, followed closely by pharmacological interventions. The sizable study of psychological interventions for pediatric chronic pain is promising given its prioritization among patients, family members, and treating health care providers^7^ but stands in stark contrast to the generally poor access to specialized multidisciplinary pediatric chronic pain intervention^17^ or mental health treatment.^82^ Three reviews focused on the remote or computerized delivery of psychological interventions.^47,56,75^ Far fewer systematic reviews examined interdisciplinary interventions despite this being the recommended approach to chronic pain management,^83^ followed by reviews of other interventions such as alternative diets, herbal supplements, and surgeries. The fewest reviews examined physical interventions, which highlights this as a key area for further research given its prioritization by patients and families,^7^ as well as the evidence for multimodal interventions, of which physical interventions are included. Possible contributing factors for less evidence in these areas could be their greater difficulty in studying with traditional clinical trial methodologies and fewer professionals in areas outside of medicine and psychology with advanced training to conduct research.

The largest proportion of reviews included diverse pediatric chronic pain populations. This suggests the applicability of many interventions across types of chronic pain and aligns with an all-encompassing primary chronic pain diagnosis.^2^ Reviews with medically complex children and adolescents were largely absent, with the exception of cerebral palsy.^80^ No reviews obviously addressed interventions for children with cognitive or intellectual disabilities or those who are nonverbal, which is of concern given their greater risk for undertreated and poorly recognized pain.^84^ When reviews focused on single patient groups, headaches and migraines or abdominal pain were the most common, possibly reflecting their higher prevalence rates.^85^ Reviews of interventions for pediatric migraines and headaches offered unique contributions and alignment with patient-oriented priorities not well addressed by other evidence, including a focus on prevention (prophylaxis) and management of acute pain flares. Only one review focused on interventions in the emergency department.^63^ This is of great relevance given the high frequency with which children with chronic pain seek care in the emergency setting,^63^ its high economic cost, the use of opioids to treat acute pain, and the potential for interdisciplinary care to reduce utilization of emergency care.^86,87^ Other reviews largely addressed interventions in outpatient or community clinics or within tertiary care centers.

With regards to intervention impact, all reviews addressed the PedIMMPACT^29^ recommended outcome of pain intensity, with fewer reporting on outcomes related to physical (disability, mobility), emotional (anxiety, depression), and role functioning (school attendance) or quality of life. Fewer still reported outcomes of treatment satisfaction or adverse events, with very little about sleep or economic factors. This reflects a neglect of outcomes identified as relevant by patients, family members, and treating health care providers, such as self-efficacy, participation in meaningful activities, social roles and relationships, vocational planning, concentration, acceptance, and resilience.^7,88^ Although almost half of reviews addressed emotional functioning, many excluded children with co-occurring primary mental health disorders. Thus, these reviews effectively omitted a large proportion of children with chronic pain with mental health concerns^89^ and decreased the relevance of available evidence to the identified patient-oriented priority about how co-occurring chronic pain and mental health can be effectively addressed.^7^ Given that the estimated annual incremental costs of treating an individual with chronic pain are CA$1742 per person, costing billions to society overall,^86^ there is a clear need to better demonstrate the economic benefit of evidence-based interventions to guide policymakers and decision makers. Though this review focused on previously recommended key outcome categories for clinical trials of interventions for pediatric chronic pain,^29^ we note that this approach is likely to miss all outcomes included in the systematic reviews, clinical practice guidelines, or the original studies they include. Other than physical and psychological interventions, less than 10% of included reviews addressed any of the other top ten patient-oriented priorities. The movement toward patient engagement and partnership in health research offers a great opportunity to lessen the divide between existing intervention studies and outcomes and that of patient priorities.^13,90,91^ Effectiveness-implementation hybrid research designs are gaining traction to enhance public health impact through efficient, feasible, sustainable, and widespread adoption of studied treatments.^13,92,93^

### Limitations

Several limitations warrant mention in considering the above presented evidence. First, this review and evidence and gap map included published systematic reviews and clinical practice guidelines only. A comprehensive review of all original intervention studies in pediatric chronic pain would be a phenomenal undertaking and beyond the scope and resources available. However, it is possible, if not likely, that additional original studies exist with relevance to identified patient-oriented research priorities that are not captured here (see interventions to educate teachers^94^ and health care providers^95^ about pediatric chronic pain, for example). This suggests that the current review overlooks areas or priorities where systematic reviews have not yet been conducted and/or in research areas less likely to rely on randomized controlled trials or other traditional treatment study designs. The patient-oriented priorities with minimal systematic review evidence shown here would likely benefit from quality systematic reviews of original studies.

### Conclusions

This systematic review reveals the great amount of contemporary evidence synthesis that has been conducted to identify effective multimodal interventions for pediatric chronic pain to date. Creation of an evidence and gap map identifies the availability of sufficient quality evidence to guide the development of evidence-informed policies and additional practice guidelines, most notably regarding psychological and pharmacological interventions to improve children’s pain and quality of life and across physical, emotional, and role functioning domains. Despite this success, the numerous obvious evidence gaps in the top patient-oriented research priorities and treatment outcomes in pediatric chronic pain should be noted by health research funders and researchers to guide prioritization of funds, as well as study aims and design.

## Supplementary Material

Supplemental Material
