# Supplementary material for: Mapping the evidence and gaps of interventions for pediatric chronic pain to inform policy, research, and practice: A systematic review and quality assessment of systematic reviews
Source: Can J Pain. 2020 Jun 19;4(1):129–48. doi: 10.1080/24740527.2020.1757384 (PMC7951164; doi:10.1080/24740527.2020.1757384)
Supplement: Supplemental Material [file UCJP_A_1757384_SM0473.docx]

Sample Search Strategy for Ovid MEDLINE(R) and Epub Ahead of Print, In-Process & Other Non-Indexed Citations and Daily

| **#** | **Searches** |
| --- | --- |
| 1 | exp Pain/ |
| 2 | (pain or pains).tw,kf. |
| 3 | ("abnormal feeling of chest" or allodynia or allodynias or alveolalgia or alveolalgias or aphagia or aphagias or arthralgia or arthralgias or back ache or back aches or backache or backaches or backpain or backpains or breast tenderness or burning sensation or burning sensations or cephalalgia or cephalalgias or cephalgia or cephalgias or cephalodynia or cephalodynias or cervicalgia or cervicalgias or cervicodynia or cervicodynias or cheiragra or chest discomfort or chiragra or chiragras or colic or cruralgia or cruralgias or cystalgia or cystalgias or dorsalgia or dorsalgias or dry socket or dry sockets or dysmenorrheal or dysmenorrheas or dysmenorrhoea or dysmenorrhoeas or dyspareunia or dyspareunias or dysuria or dysurias or earache or earaches or ear ache or ear aches or headache or headaches or head ache or head aches or hemicranias or hyperalgesia or hyperalgesias or hyperalgia or hyperalgias or hyperpathia or hyperpathias or hypoalgesia or hypoalgesias or lumbago or lymphadenopath* or mastalgia or mastalgias or mastodynia or mastodynias or menstrual cramp or menstrual cramps or metatarsalgia or metatarsalgias or muscle soreness or myalgia or myalgias or myodynia or myodynias or neckache or neckaches or neck ache or neck aches or neuralgia or neuralgias or neuralgy or neurodynia or neurodynias or odontalgia or odontalgias or odynophagia or odynophagias or orchialgia or orchialgias or otalgia or otalgias or painful breathing or painful defecation or painful defecations or painful erection or painful erections or painful micturition or painful micturitions or painful respiration or painful respirations or painful scrotum or painful testis or paroxysmal nerve pain or paroxysmal nerve pains or pelipathia vegetativa or pelvic syndrome or pelvis syndromes or pharyngalgia or pharyngalgias or piriformis muscle syndrome or piriformis syndrome or piriformis syndromes or polyarthralgia or polyarthralgias or precordialgias or prostatalgia or prostatalgias or prostatodynia or prostatodynias or psychalgia or psychalgias or rachialgia or rachialgias or sciatica or sciaticas or slit ventricle syndrome or slit ventricle syndromes or sore throat or sore throats or stomatodynia or stomatodynias or stomatopyrosis or stranguria or strangurias or strangury or thoracic discomfort or thorax discomfort or throat ache or tooth ache or throat aches or tooth ache or tooth aches or toothache or toothaches or vulvodynia or vulvodynias).tw,kf. |
| 4 | or/1-3 |
| 5 | (chronic* or recur or recurs or recurr* or persist* or continue* or continual).tw,kf. |
| 6 | 4 and 5 |
| 7 | chronic pain/ |
| 8 | 6 or 7 |
| 9 | (infan* or newborn* or "new born*" or neonat* or baby* or babies or toddler* or minors* or boy or boys or boyfriend or boyhood or girl* or kid or kids or child* or adolescen* or juvenil* or youth* or teen* or "under* age*" or pubescen* or pediatric* or paediatric* or peadiatric* or prematur* or preterm*).mp. or school*.tw. |
| 10 | 8 and 9 |
| 11 | Meta-Analysis as Topic/ |
| 12 | (meta analy* or metaanaly).tw. |
| 13 | Meta-Analysis/ |
| 14 | (systematic adj (review$1 or overview$1)).tw. |
| 15 | exp Review Literature as Topic/ |
| 16 | (cancerlit or cochrane or embase or psyclit or psychlit or psycinfo or psychinfo or cinahl or cinhal or "science citation index" or bids).ab. |
| 17 | ("reference list*" or bibliograph* or "hand search*" or "relevant journal*" or "manual search*").ab. |
| 18 | ("selection criteria" or "data extraction").ab. |
| 19 | Review/ |
| 20 | 18 and 19 |
| 21 | or/11-17,20 |
| 22 | Comment/ or Letter/ or Editorial/ |
| 23 | 21 not 22 |
| 24 | 10 and 23 |
| 25 | remove duplicates from 24 |
